# Factors Influencing the Utilization of Voluntary Counselling and Testing Services among University Students in Kenya

**DOI:** 10.5539/gjhs.v6n4p84

**Published:** 2014-04-10

**Authors:** Rose Mwangi, Peter Ngure, Moses Thiga, Jane Ngure

**Affiliations:** 1 School of Communication, Languages and Performing Arts, Daystar University, Kenya; 2 School of Science, Engineering and Health, Daystar University, Kenya; 3 ICT Department, Kabarak University, Kenya; 4 Counseling Psychology Department, Africa Nazarene University, Kenya

**Keywords:** higher learning institutions, risky sexual behaviour, stigma, HIV testing, VCT

## Abstract

Voluntary Counselling and Testing (VCT) is recognized as a critical component of effective HIV prevention initiative and has therefore been promoted nationally and within universities in Kenya. Upon successful counselling and testing those found to be HIV negative are informed to take the necessary measures to protect themselves while the infected are advised to start the required medication. This study examined the attitudes toward VCT services among university students in four Kenyan universities. 980 students filled self administered questionnaires. Results showed that 38.5% of the subjects had tested for HIV in the last 12 months and students (55.8%) felt less susceptible to HIV infection. Findings from a factor analysis revealed that the intention to seek the services was associated with five attitude subscales that were ranked as follows (i) people’s and personal concerns, (ii) friends concerns, (iii) value of testing, (iv) confidentiality and support, and (v) perceived susceptibility. The first three items are associated with stigma which was evidenced in the subjects’ report that admitting that one should test for HIV would imply that one has engaged in immoral behaviour. Secondly, subjects felt that their friends would look down on them if they tested for HIV. Knowing the students’ attitudes will therefore assist in the development of appropriate VCT interventions that will promote HIV testing and behaviour change.

## 1. Introduction

Testing for HIV antibodies is an important component of prevention and intervention programmes designed to curb the spread of HIV infection, especially in Kenya which has the highest national HIV prevalence in any country outside South Africa (NACC & NASCOP, 2012). The 1.6 million Kenyans living with HIV in 2011 represented almost four-fold increase over the 400,000 people living with HIV in 1990 (NACC & NASCOP, 2012). More than 91,000 Kenyan adults became HIV infected in 2011 (NACC & NASCOP, 2012). It is estimated that the number of people infected with HIV in Kenya will continue to increase and may approach 1.8 million by 2015 (NACC & NASCOP, 2012). The HIV pandemic in Kenya is complex as it continues to evolve among different populations, which makes it difficult to predict. Young adults between ages 15 to 35 represent 38% of the national population but are believed to make up more than 60% of new HIV infections in Kenya. The HIV virus is mainly transmitted through heterosexual intercourse that accounts for 77% of all new infections (NACC, 2009; NACC & NASCOP, 2012).

University going students, the main focus of this paper, are among the risky groups in Kenya (EAC/AMREF, 2010; Gitonga, Sinyard, & Gachuiri, 2012; Magu, Wanzala, Mutugi, & Ndahi, 2012; NACC & NASCOP, 2012). Studies indicate that a high proportion (68%) of university students, ages 17 – 24 years old, in Kenya are sexually active with peaks in the first and second years of study (EAC/AMREF, 2010; Gitonga et al., 2012; Othero, Aduma, & Opil, 2009). Mean age at sexual debut is 16.5 years, which is strongly correlated with increased risk of HIV infection especially among the young women in Kenya (Gitonga et al., 2012; KNBS, 2010). University students are therefore an important constituency in the interventions against HIV and AIDS as many might be entering university before they are sexually active and yet they fall within the age bracket where HIV infection peaks. The students also represent future leaders and are the economic backbone of Kenya, which hopes to attain middle class economy by the year 2030 (NACC & NASCOP, 2012)

Universities constitute a potentially fertile breeding ground for HIV and AIDS as previous studies indicate. Universities bring together in close physical proximity devoid of systematic supervision a large number of young adults at their peak years of sexual activity and experimentation (Katahoire, 2004). Students experience increased freedom from parental and school control. Some students like the first year females are coerced and subjected to sexual harassment (Adam & Mutungi, 2007). There are reports of multiple sexual partners among the students. Also concerns of sexual relationships between female students and male faculty, with the exchange of sex for grades, not to mention prostitution among the females as a means of self-sustenance (Adam & Mutungi, 2007; EAC/AMREF, 2010). All these coupled with environmental influences: availability of alcohol, urban life style of discos and low social economic status, are some of the factors that facilitate risky behaviour among the students (Kamaara, 2005; Katahoire, 2004).

The students are also vulnerable to HIV infection as condom use is inconsistent or rare especially during the first sexual episode (Othero et al., 2009; Museve, George, & Labongo, 2013). Free condoms available in the university dispensers are less respected than those purchased. Female students do not hold their male counterparts with high esteem if they use condoms from the campus dispensers, which call for purchase of condoms even for the economically challenged students (Adam & Mutungi, 2007; KNBS, 2010; NACC & NASCOP, 2012; Magu et al., 2012). Lack of condom use is associated with HIV and other sexually transmitted infections.

To avert a HIV crisis, universities in Kenya in partnership with other organizations among them, the National AIDS Control Council (NACC), Liverpool VCT Care and Treatment (LVCT) and I Choose Life, have taken up the fight against the spread of HIV infection by establishing HIV/AIDS Control Units (ACU). ACU provides an institutional framework for addressing HIV, facilitates peer education aimed at appropriate behaviour change practices and positive living. They acquire and distribute condoms within campus and formulate HIV policies. They host annual HIV counselling and testing week where students and staff are encouraged to know their HIV status (Kenyatta University, 2013; UON, 2003; DAYSTAR, 2007; EAC/AMREF, 2010). VCT services in Kenya are also available in community based sites and health facilities. VCT promotional campaigns are carried out during major events like the 2010 world cup tournament where testing was encouraged as people watched the game in public places (KNBS, 2010)

Core principles of HIV testing and counselling include individuals receiving VCT give informed consent, which makes testing voluntary. VCT services are confidential, meaning anything discussed between the VCT provider and client is not disclosed to a third party. HIV test is accompanied by pre-test information and post-test counselling including referrals to appropriate services (NASCOP, 2010).

**Figure 1:**
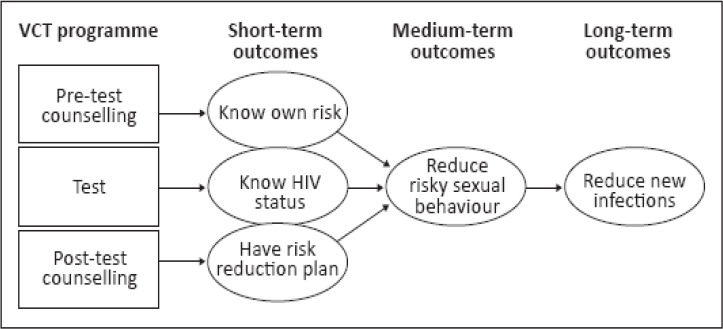
Programme theory for VCT interventions (Anderson & Louw-Potgieter, 2012)

As noted in South Africa, VCT services aim at achieving short, medium and long-term outcomes relating to HIV/AIDS risks and status of the individual as shown in Figure 1 (Anderson & Louw-Potgieter, 2012). Short-term outcomes relate to knowledge, acquisition of HIV, how it is transmitted and to know ones HIV status. Medium-term outcomes are associated with changes in risky sexual behaviour and safer sexual behaviour. Those found negative are encouraged to protect themselves by using condoms and by having one faithful partner, while those infected are given support and the necessary treatment (Anderson & Louw-Potgieter, 2012; NASCOP, 2010). Ultimately, long term outcomes should manifest a reduction of new infections.

### 1.1 The Problem

Universities in Kenya have worked to equip students with knowledge on HIV transmission and prevention. University going students in Kenya are knowledgeable about HIV and they started learning about the infection back in their elementary and secondary school education. That explains why all Kenyans are reported to be knowledgeable about HIV (NASCOP, 2010). Unfortunately having HIV knowledge has not translated to behaviour change. Students are reported to engage in risky sexual behaviour that range from multiple sexual partners to inconsistent condom use (Museve et al., 2013; Othero et al., 2009). Given that the University students are sexually active, they need VCT services so as to know their HIV status. National statistics indicate that only 48% Kenyans know their HIV status. The utilization of VCT services has continued to decline to as low as 14.4% (NASCOP, 2009; NACC & NASCOP, 2012). The rate of HIV counselling and testing among the youth, who include university going students, is equally low (KNBS, 2010; NACC & NASCOP, 2012).

Universities in Kenya show inconsistencies in VCT usage. For example, findings from a study of six Kenyan universities showed that a majority (63.6%) of the students had ever tested for HIV (EAC/AMREF, 2010), few (32%) students had tested in Maseno University (Othero et al., 2009) and only 18.4% had tested in Mt Kenya University HIV open day carried out by Liverpool VCT (Museve et al., 2013). These inconsistent testing patterns contradict previous studies that indicated HIV testing was higher among those with at least secondary education since they understood the importance of HIV testing (Barker et al., 2012; Kalichman & Simbayi, 2004; KNBS, 2010). University students are not ignorant of the benefits of VCT yet few have utilized the services (Anderson & Louw-Potgieter, 2012; KNBS, 2010; NASCOP, 2009; Peltzer, Nzewi, & Mohan, 2004).

It was therefore necessary to investigate the usage of VCT services among the students guided by the following research questions: 1) what were the HIV testing patterns among university students in Kenya. 2) What was the students’ level of perceived susceptibility to HIV and AIDS 3) What were the students’ attitude and beliefs towards VCT services in Kenya 4) And what were the factors influencing the students’ utilization of the VCT services. Although different HIV studies have been carried out on the general Kenyan population including the high school and out of school youth, studies specific to institutions of higher learning are few (EAC/AMREF, 2010). There are also limited publications of HIV related data touching on university students in Africa and indeed in Kenya. University going students are the foundation of a growing society, their energy, inventiveness, character and orientation define the pace of development of a nation (EAC/AMREF, 2010; UNAIDS, 2008). Findings of this study will assist policy makers and subsequently the universities in planning effective VCT interventions among the students.

## 2. Methodology

### 2.1 Research Design

The study was a quantitative survey which used questionnaires among students in selected universities.

### 2.2 Sample Size and Sampling Method

Four universities were purposively selected for the study. They included two public universities, namely University of Nairobi (UON) and Egerton University, and two private universities, namely Daystar University, and African Nazarene University. The University of Nairobi was selected for the study because of its location in Nairobi City, a typical urban environment where cost of living is high and there is easy access to entertainment facilities. Egerton University was selected because of its sub-urban location in Kenya’s Rift Valley. Both Daystar and African Nazarene universities were among the first established private institutions in Kenya. Daystar is located in the outskirts of Nairobi at the foot of the Lukenya Hills, a plain land that houses a host of African wildlife. The area has little entertainment joints and students seeking entertainment have to travel to Nairobi City over the weekends. African Nazarene is located in a rapidly growing town near Nairobi City and students can easily access the city.

All students in the selected universities were eligible for the study and so questionnaires were distributed to any willing participant. A total of 980 participants filled the questionnaires out of the expected 1300, which represented 75.4% of the targeted sample. These comprised of 276 students from the University of Nairobi, a response rate of 69%. 308 students from Egerton University, response rate of 77%. 208 students from the African Nazarene University, response rate of 83.2%. And 188 students from Daystar University, a response rate of 75.2%. This number was well above the 300 minimum requirements for an exploratory factor analysis approach that was adopted for this study (Demo et al., 2012).

### 2.3 Measurements

A self-administered closed-ended questionnaire was used to collect the data. Attitudes towards HIV testing were measured using a five point likert scale comprising of 41 items. The 5-point likert scale was modified from a scale developed to measure attitudes towards HIV antibody testing (Boshamer & Bruce, 1999; Peltzer & Mpofu, 2002). By identifying an individual’s attitude about HIV testing, concerns about the testing process can be highlighted and this helps in the development of appropriate interventions within the targeted group (Boshamer & Bruce, 1999). The five options on the scale and their scores were as follows; strongly disagree (1), disagree (2), neutral (3), agree (4) and strongly agree (5). A number of barrier items were also included and were scored in reverse. There were additional items to capture additional information such as demographic data (4 items), sexual behaviour and perceived susceptibility to HIV (4 items), HIV testing behaviour (4 items). A pilot study was conducted in order to establish the reliability of the instrument. A cronbach alpha score of .81 was obtained indicating that the instrument had a high internal consistency.

#### 2.3.1 Data Analysis

Data was analyzed using Statistical Package of Social Sciences (SPSS) software. The software was used to generate descriptive statistics and work out the factor loadings required to identify the items influencing the students’ utilization of VCT services.

### 2.4 Ethical Considerations

Permission for the project was obtained from Kenya Ministry of Education and approval for the study was sought from Daystar University Research, Publication and Consultancy Department. The data collected was handled confidentially and the respondents were not required to identify themselves when filling the questionnaire

## 3. Results

### 3.1 Demographics

The 980 respondents who adequately filled the questionnaires were represented by 47.7% male and 52.3% female. The average age of the respondents was 22 years. A majority (88.8%) of the respondents were 19 – 24 years, 2.3% were between 17 - 18 years and 8.9% were 25- 42 years. On marital status a majority (93.2%) were single, a few were married (5.0%) and divorced (1.8%). Distribution across year of study were, 28.2% first years, 33% second, 23.2% third, 14.8% fourth and 0.9% fifth years. Majority (87.8%) were full-time students and 12.2% part-time.

### 3.2 Voluntary Counselling and Testing

Subjects who had tested for HIV in the last 12 months were 38.5%. University of Nairobi led with 41.7%, followed by Egerton (39%), Daystar (37.8%) and African Nazarene (34.1%). Students (71.7%) who had not tested for HIV expressed the desire to test in the next 12 months but 28.3% had no intention of testing in the future. The subjects (82%) felt that one is free to test for HIV at any time, before marriage (34.1%) and when one has multiple sexual partners (32.7%). Reasons why one should test for HIV: because it is beneficial (89.4%), to know HIV status (89.7%), avoid transmission of HIV virus (34.7%), and protect self from infection (27.8%).

### 3.3 General Attitudes to VCT

On the general attitudes to VCT, majority (91.9%) of the subjects felt that it was extremely useful to test for HIV and 83.3% considered VCT to be extremely important. However, a majority (44.1%) disagreed with this statement: ‘*testing and counselling for HIV is a pleasant experience*,’ only 28.6% agreed with the statement. Some of the subjects (45.3%) considered going for HIV counselling and testing extremely frightening. 72.4% disagreed that their schooling would be in danger if the university found out that they had tested for HIV. On VCT confidentiality 41.3% felt HIV results were accurate, 49.9% felt health providers can be trusted with their counselling and test information. Some 52.1% felt that they could comfortably talk to a counsellor about personal behaviours that might place them at risk for HIV infection.

### 3.4 Susceptibility to HIV Infection

Most (68.5%) of the subjects disagreed with the statement, ‘*There is a possibility that I have HIV and AIDS’*, only 10% agreed with the statement. Equally more (59%) subjects disagreed with the statement, *‘I may have had sex with someone who was at risk for HIV and AIDS’*, 18.9% agreed with the statement. To the statement, *‘I am at risk for HIV and AIDS’*, 39.9% disagreed with the statement, while 36.8% agreed that they were at risk

### 3.5 Items Influencing students’ Utilization of VCT Services

Like Boshamer and Bruce (1999) factor analysis using principal components and the varimax rotation was used to identify factors influencing the utilization of VCT services among the students sampled. Five factors with an eigen value of above 1.5 were identified using the Scree test. The identified factors accounted for a total of 32.8% of the variance in scores.

The cut-off factor loadings acceptable in this study were reached using Norman and Streiner’s (1994) formula for calculating the minimum loading when the sample size, N, is 100 or more: Min FL = 5.152/SQRT(N-2). In this study N=980 respondents, which resulted in a minimum 0.165 as the minimum cut-off. It is observed that common social science practice uses a minimum 0.3 or 0.35 (Rummel, 1970; Peltzer & Mpofu, 2002). However, loadings less than 0.4 are generally termed as ‘weak’, those more than 0.6 are ‘strong’ especially in a likert scale. Thus, 0.4 was considered as the minimum cut-off in this study and a total of 32 items, presented in Table 1 met this criteria.

**Table 1:** Items and factor loadings for the HIV antibody testing attitude scale

Factor	Loading
**Factor I. People’s and personal concerns**	
I consider going for HIV counselling and testing extremely frightening	.462
People assume that everyone who is tested for HIV is infected with HIV	.416
Testing and counselling for HIV is a pleasant experience	.503
It would be embarrassing to get tested for HIV	.497
I would not consider getting a HIV test because I would be asked about things I have done that could get me into trouble	.467
I consider going for HIV counselling and testing extremely humiliating	.479
Testing and counselling for HIV is extremely intimidating	.568
I could easily discuss HIV antibody testing with my family	.420
People would assume I have HIV if I decided to get tested	.473
Admitting that you should be tested for HIV means that you have engaged in immoral behaviour	.554

**Factor II. Friends concerns**	
My friends would not look down on me if I were tested for HIV	.533
My parents would be upset if they knew I was planning to get tested for HIV	.441
I am afraid that if I were tested for HIV, my name would go into public records	.445
My friends would look down on me if I were tested for HIV	.680
I am afraid someone would find out I was tested for HIV	.486
I would be embarrassed if my friends found out I had decided to have an HIV test	.446
My friends would treat me badly if I were tested for HIV	.645
My friends would not treat me any differently if I were tested for HIV	.484

**Factor III. Value of HIV testing**	
It is extremely useful to test for HIV	.528
Anyone who is tested for HIV is disgusting	.441
Testing and counselling for HIV is extremely beneficial	.559
Anyone who is tested for HIV is dirty	.472
I do not have time to get an HIV test	.430
I consider going for HIV counselling and testing extremely important	.479

**Factor IV. Confidentiality and support**	
HIV test information is kept very confidential by the medical staff who do the testing	.643
My friends would support my decision to get a HIV test	.415
HIV tests give accurate results	.535
I trust the HIV test counsellor and nurses to keep my information confidential	.671
HIV counselling and testing is not really confidential	.454

**Factor V. Perceived susceptibility**	
There is a possibility that I have HIV and AIDS	.666
I may have had sex with someone who was at risk for HIV and AIDS	.668
I am at risk for HIV and AIDS	.656

The first factor (eigenvalue = 6.90) accounted for 15.7% variance in the responses and contained items concerned largely with how people might react to one’s HIV testing, and how an individual feels towards HIV testing. Items such as; ‘*Testing and counselling for HIV is extremely intimidating’* and *‘Admitting that you should be tested for HIV means that you have engaged in immoral behaviour’* loaded highly in this factor,

The second factor (eigenvalue = 2.2) accounted for 5.0% of the variance in responses and included items that were mainly concerned with how friends might react to HIV testing. Items that loaded highly included; ‘*My friends would treat me badly if I were tested for HIV’* and ‘*My friends would look down on me if I were tested for HIV’*.

The third factor (eigenvalue = 2.0) accounted for 4.6% variance in the responses and included items that were mainly concerned with importance of HIV testing. Items such as; ‘*It is extremely useful to test for HIV’* and *‘Testing and counseling for HIV is extremely beneficial’* scored highly.

The fourth factor (eigenvalue = 1.7) accounted for 4.0% variance in the responses and contained items mainly concerned with confidentiality and support. Items that loaded high included; ‘*HIV test information is kept very confidential by the medical staff that do the testing’, ‘HIV tests give accurate results’, ‘I trust the HIV test counsellor and nurses to keep my information confidential’*.

The fifth factor (eigenvalue = 1.5) accounted for 3.5% variance in the responses and contained items mainly concerned with perceived susceptibility. The following items loaded highly; ‘*There is a possibility that I have HIV and AIDS’, ‘I may have had sex with someone who was at risk for HIV and AIDS’* and *‘I am at risk for HIV and AIDS’*.

## 4. Discussions

In recognition of the role played by VCT services in mitigating the negative impact of HIV and AIDS, this study sought to establish the attitude towards VCT services among university students and their perceived susceptibility to the HIV virus, after which factors that influence the use of VCT services where extracted through factor analysis.

The findings in the study indicated that the acceptability of VCT was low as more than half of the students had not had VCT in the last 12 months. Both public and private universities had almost the same relatively low patterns of HIV testing. Universities reported Low VCT uptake despite the enormous promotions within the campuses (Daystar, 2007; KU, 2013; Mwiria, 2007; UON, 2003). The students had also exhibited confidence in their universities’ support in seeking VCT, they were aware that one can take VCT at any time; and it was an important and beneficial exercise.

Low VCT uptake has previously been reported in Kenyan Universities, for example: in Maseno University (32%) (Othero et al., 2009), and Mt Kenya HIV open day (18.4%) (Museve et al., 2013). Like this study the VCT uptake is low which means that there are some factors that influence the uptake of VCT among the students. It appears that even among medical students VCT uptake is not a hundred percent as observed in a study in Jos University, Nigeria. The study showed that only about half (50.7%) of the participants had VCT although they were aware of the VCT services at the hospital and were not ignorant of the importance of knowing one’s HIV status (Daniyam, Agaba, & Agaba, 2010).

Some other studies in Kenyan universities showed a more receptive attitude to VCT uptake, for example, in Kimathi University 52.6% of the participants had ever tested for HIV (Gitonga et al., 2012); findings from six Kenyan universities showed that 63.6% had ever tested but 36.3% had never tested for HIV (EAC/AMREF, 2010). The figures were higher than those in this study. The discrepancy in the findings may be explained by differences in the questionnaire. This study sought whether respondents had tested in the last 12 months while the other studies sought to find out if the participants had ever tested. It appears that respondents may have tested at least once.

This study sought the last 12 months because HIV and AIDS have a long incubation period. Six months after becoming infected, a person can test negative while carrying the virus and can easily pass the virus to others without knowing it (Coon & Mitterer, 2010). Given the risky behaviour of the students, it would be expected that the students seek VCT more often so as to know their status. VCT is provided free in the universities, the campuses promote a VCT week once a year and there are policies that support VCT (Daystar University, 2007; University of Nairobi, 2003).

At the same time only few students thought that one should seek VCT services so that if one is positive, they can take measures to avoid transmitting the HIV virus to others. Again, less than thirty percent thought that one should seek VCT services so as to protect themselves from HIV infection. The students’ attitude seemed to contradict the idea that seeking VCT was an important means of preventing the transmission of HIV virus (Kalichman & Simbayi, 2003; Alemu, Abseno, Degu, Wondmikun, Amsalu, 2004; Anderson & Louw-Potgieter, 2012; UNGASS, 2010). It seems that university students might not have fully embraced the need to seek VCT services as a preventive measure against the spread of HIV virus although they agreed testing was a means of knowing ones status.

Like previous studies, participants who had not tested for HIV planned to test in the next 12 months. Subjects who had not tested in Maseno University (70.8%) and the study among six Kenyan universities (84.1%), all intended to test for HIV in future (Othero et al., 2009; EAC/AMREF, 2010). The Theory of Reasoned Action suggests that the main determinant of any behaviour is the intention to perform that behaviour (Ajzen & Fishbein, 1980). Unfortunately, research shows that desire to test for HIV antibodies does not always mean actual testing (Museve et al., 2013). Twenty eight percent of the participants in this study were at great risk of HIV infection as they were not willing to undergo VCT. About a quarter of the Mt Kenya students were also not willing to undergo VCT in the future, with 53.6% citing that the uptake was not an easy exercise. Not testing for HIV is a high risk as previous national survey in Kenya showed that 28% of adults who had not tested and were not aware of their HIV status mistakenly believed themselves to be HIV negative (NASCOP, 2009, NACC & NASCOP, 2012).

Most of the respondents felt that they were less susceptible to HIV infection as many disagreed that there was a possibility that they might be infected with HIV and AIDS. The students’ confidence might have been pegged to the fact that many believed that they had not had sex with someone at risk of HIV as other studies have shown (Pappas, Yujiang, & Khan, 2011). These findings are similar to responses from students in other studies: for example, a study at an urban-based university in South Africa showed that students tend to distance the HIV virus from themselves and people surrounding them, they believe they cannot be infected (Shefer, Strebel & Jacobs, 2012); among Malawian first year students, 68% perceived that they were not at risk of acquiring HIV (Ntatal, Muula, Siziya, & Kayambazinthu, 2008); and 80% male and female at another South African university reported that they had never worried about HIV infection (Peltzer, 2005). A past national survey in Kenya indicated that the personal belief that one was not at risk for HIV was the single most important reason non-testers for HIV gave for having failed to be tested (NASCOP, 2009).

Individuals vary widely in their feeling of personal vulnerability to a health condition (Janz & Bekky, 1984). Some people (low perceptible) believe it is very unlikely that they would contract a particular disease or health related condition (Infante, Rancer, & Womack, 2003; Rosenstock, 1961). Still others who are moderate in their perceptions of susceptibility may admit to the statistical possibility of its occurrence and see the probability of them suffering the condition. In the case of HIV and AIDS, some people tend to be concerned about the increase of the scourge in the general public but they hardly think of the infection affecting them (Freimuth et al., 1990). Thus the infection is seen as other people’s problem but not theirs. Those who feel more susceptible are likely to feel threatened by the health condition, thus will be keen to know their HIV status unlike those who feel less susceptible (Janz & Bekky, 1984; Rosenstock, 1961). However, perceived susceptibility alone does not always seem to lead to HIV testing as shown in a study among Maseno University students in Kenya. Students perceived higher susceptibility to HIV as 74.3% felt that there was a likelihood of them being infected with HIV virus based on their previous risky sexual experiences, but only 32% reported having undergone HIV tests (Othero et al., 2009).

Results from the factor analysis revealed that having gone for voluntary counselling and HIV testing and the intention to seek the services was associated with five attitude subscales that were ranked as follows: people’s and personal concerns, friends concerns, value of testing, confidentiality and support, and lastly perceived susceptibility. Confidentiality, support and perceived susceptibility were weakly associated with the motivation to test for HIV. A previous study among university students in four African countries showed that going for HIV test was associated to: general concerns, trust and support, and fears. Confidentiality and Friends concerns subscales were weakly associated with the intention to go for HIV test (Peltze, Mpofu, Baguma, & Lawal, 2002).

The most important factors were people and personal concerns, followed by friends concerns all of which are associated with stigma and discrimination. Study in the six Kenyan universities showed that there is yet no integrated and coherent response to HIV and AIDS in the higher education sector (EAC/AMREF, 2010). Although universities in Kenya have established HIV and AIDS centres, institutions surveyed still had a culture of discrimination and stigma. There is therefore need to institutionalize HIV and AIDS as a core responsibility, mobilize adequate resources, promote research and be able to break HIV and AIDS from a culture of silence to a culture of critique and openness (EAC/AMREF, 2010). HIV and AIDS differ from other forms of prejudices (racism, sexism etc) since it is infectious. Secondly, once infected, one is likely to be stigmatized by those they would have previously considered to be their own group (Stein, 2003). A stigmatized person is seen as having a ‘spoiled identity’ in relation to that group and is regarded as a deviant or abnormal (Stein, 2003). Naturally, this will affect the uptake of VCT among young university students as nobody would like to be rejected by their peers and the public. Subjective norms, which refer to the perceived social pressure or support to perform or not to perform a required behaviour, affect the outcome of one’s behaviour (Ajzen, 2002). Individuals who are informed about the importance of the VCT also need motivation from friends and the public in order to perform the required behaviour (Boshamer & Bruce, 1999; Fisher & Fisher, 1992).

The value of HIV testing together with confidentiality and support are related to the positive or negative consequences of undertaking the VCT services. Perceived susceptibility relates to personal behaviour that makes one perceive that they could be in danger or are not in any danger of getting the HIV infection. The factor analysis eliminated family concerns as significant influence on the HIV testing patterns of the students. That might be attributed to the fact that most university students are considered mature enough to make their own decisions, and the fact that they are away in campus for many months, which can limit their family influence.

## 5. Conclusion

Considering that the young people in Kenya are at risk of contracting HIV virus and the fact that universities are reported to constitute a potentially fertile breeding ground for the virus, it is important for university students to know their HIV status. Unfortunately, the study has revealed that the provision of VCT services in campuses and holding VCT days was not a guarantee that students will seek VCT services in view of the fact that some students do not perceive themselves to be at risk of HIV infection. People and personal concerns plus friends concerns, that are associated with stigma, were identified as the main factors that influence the usage of VCT. It is therefore important to reduce the levels of stigma and discrimination in Kenyan universities and encourage students to seek VCT services within and outside their campuses. Universities should also promote peer to peer counselling and accompany each other when seeking VCT services.
